# The presence of acquired enamel pellicle changes acid-induced erosion from dissolution to a softening process

**DOI:** 10.1038/s41598-017-11498-1

**Published:** 2017-09-07

**Authors:** Mahdi Mutahar, Guy Carpenter, David Bartlett, Matthew German, Rebecca Moazzez

**Affiliations:** 1 Salivary Research, Mucosal and Salivary Biology Division, King’s College London Dental Institute, London Bridge, London, SE1 9RT United Kingdom; 2 Tissue Engineering and Biophotonics, King’s College London Dental Institute, London Bridge, London, SE1 9RT United Kingdom; 3 Centre for Oral Health Research, School of Dental Sciences, Newcastle University, Newcastle upon Tyne, NE2 4BW United Kingdom

## Abstract

Erosive wear undermines the structural properties of enamel resulting in irreversible enamel loss. A thin protein layer formed from natural saliva on tooth surfaces, acquired enamel pellicle (AEP), protects against erosive wear. The exact components in saliva responsible for such protection are not yet known. We prepared three solutions containing different components: proteins and ions [natural saliva (NS)], minerals with no proteins [artificial saliva (AS)] and neither proteins nor ions [deionised water (DW)]. To assess the protection of the three solutions against citric acid enamel erosion, enamel specimens were immersed in the corresponding solution for 24 h. All specimens were then exposed to five erosion cycles, each consisted of a further 30 min immersion in the same solution followed by 10-min erosion. Mean step height using a non-contacting profilometer, mean surface microhardness (SMH) using Knoop microhardness tester (final SMH), and roughness and 2D profiles using atomic force microscopy were measured after five cycles. The final SMH values were compared to the starting values (after 24 hr). NS group had significantly less tissue loss but greater SMH change (P < 0.0001) than AS and DW groups. Specimens in NS were softer and rougher (P < 0.001) but less eroded than specimens in AS and DW.

## Introduction

Erosive wear has become a prevalent oral health problem, affecting an increasing proportion of the population worldwide^1^. Erosive wear can be influenced by many factors, including biological factors, particularly saliva and the acquired enamel pellicle (AEP), which may have an important role in the prevention and/or progression of erosion^2–6^. However, the difficulty of modelling dental erosion *in vitro* has led to many conflicting reports and much confusion in this field.

Natural saliva (NS) and artificial saliva (AS) (which contains the minerals but not the proteins of saliva) have been used in *in vitro* erosive wear models to assess the demineralisation/remineralisation process of erosive wear. NS plays an important role in maintaining tooth integrity, as demonstrated by severe dental disease in xerostomic subjects lacking saliva^7^. Saliva is supersaturated with minerals such as calcium, phosphate and fluoride, which help maintain the physical and chemical integrity of the tooth structure^8, 9^, but also contains more than 1,000 different proteins^10^. Selective adsorption of a specific subset of these salivary proteins to the enamel surface leads to the formation of a thin layer, free of bacteria, on oral hard tissues known as the AEP. The AEP is composed of proteins and other components such as glycoproteins, lipids and several enzymes^11, 12^. Formation of the AEP starts moments after brushing, and equilibrium saturation reaches its maximum after a time period between 30 min to 2 h^13–15^. The composition of AEP provides it with physical characteristics such as viscoelasticity, which promotes the formation of a partial barrier against acid diffusion^16–18^. Some studies have related the protective effects of the AEP to its mineral content^19–21^, whereas others have attributed these effects to its protein components^12, 22–24^. In reality, it is likely to be a combination of the two, with proteins guiding and modifying ion movements into, and out of, the tooth.

Considering the complex sources and composition of NS, a number of salivary parameters need to be considered when using it for *in vitro* studies; these parameters include time of collection, whether it is freshly collected or pooled then frozen and whether it is stimulated or unstimulated^10, 21^. Natural saliva is commonly replaced by artificial saliva in *in vitro* studies due to issues relating to collection, storage and degradation of human saliva^25–27^.

Several studies have assessed the potential use of artificial formulations in remineralising a softened erosive lesion^20, 28, 29^. The most commonly used and currently available formulations of artificial saliva^21^ fall into three types: electrolytes with carboxymethylcellulose (CMC)^28, 30^, electrolytes with mucin^25^ or electrolytes only^31^.

The aim of this *in vitro* study was to assess the role of ions and proteins in preventing acid-induced erosion by immersing human enamel specimens in NS, AS or DW and to characterise the erosive process via a range of techniques. Profilometry provides a measure of surface loss, microhardness indicates sub-surface changes and AFM measures surface changes.

## Materials and Methods

A sample size of 30 (10 per solution) was chosen based on 95% power at the 5% level with an effect size of 0.6 using ANOVA and a two-tailed test comparing step height and microhardness changes on enamel specimens immersed in the various solutions. The enamel specimens were prepared from previously extracted caries-free permanent human posterior teeth and stored in a thymol solution at 4 °C. Teeth were collected from patients attended clinics in King’s College London Dental Institute, Guy’s hospital London who were informed about the possibility of using their teeth and written consent was obtained. The experiment was carried out in accordance with the approved guidelines and regulations of the National Research Ethics Committee, London (REC ref: 12/LO/1836). All methods were performed in accordance with the relevant guidelines and regulations. The buccal and lingual surfaces of teeth were cut with a 4-inch diamond blade (Diamond wafering blade XL 12205, Benetec Ltd, London, UK) at 300 rpm and a load of 200 N using a cutting machine (Buehler GmbH, Düsseldorf, Germany). The cut specimens were placed into a custom-made silicone mould (specimen size 8 × 21.5 × 24 mm) and embedded in cold-cured acrylic resin (Oracryl, Sussex, UK). Specimens were then polished using a water-cooled rotating polishing machine (Meta-Serv 3000 Grinder-Polisher, Buehler, Lake Bluff, Illinois, USA) with continuous water irrigation to provide a surface sufficiently large and flat for analysis. Progressively abrasive grit silicon carbide papers were used, starting at 80 grit, followed by 120, 600, 1200, 2500, and 4000 grit (SiC-Paper, Struers A/S, Copenhagen, Denmark). Silicon paper of 80, 120 and 600 grit were used to remove the superficial prismatic enamel, whereas the 1200, 2500 and 4000 grit papers were used to polish the enamel surfaces. All prepared specimens were then numbered for identification and randomised, following which they were immersed in 80 ml of deionised water and ultrasonicated (Nusonics GP-70, T310) at 60 Hz for 15 min, after which they were rinsed and allowed to dry. Specimens were then taped with PVC adhesive tape to create a window of exposed enamel approximately 2 mm by 3 mm wide, with a reference area on either side.

The artificial saliva was prepared according to the protocol used by Eisenburger *et al*. (2001b)^31^. Paraffin-stimulated natural saliva samples were collected from healthy volunteers. The protocol for natural saliva collection from healthy volunteers was approved by the ethical review committee in Northampton (REC, 14/EM/0183), and written informed consent was obtained from each participant. The collected natural saliva was ice-chilled and pooled immediately after collection at −80 °C for long-term storage. Prior to use, the frozen natural saliva samples were defrosted for the same length of time (3 h) at room temperature of 22 ± 1 °C. Thawed saliva was mixed vigorously with a vortex mixer to resuspend precipitation of proteins upon thawing to avoid the loss of specific proteins of less than 14 kDa, such as statherin and/or histatins^32^.

**Figure 1:**
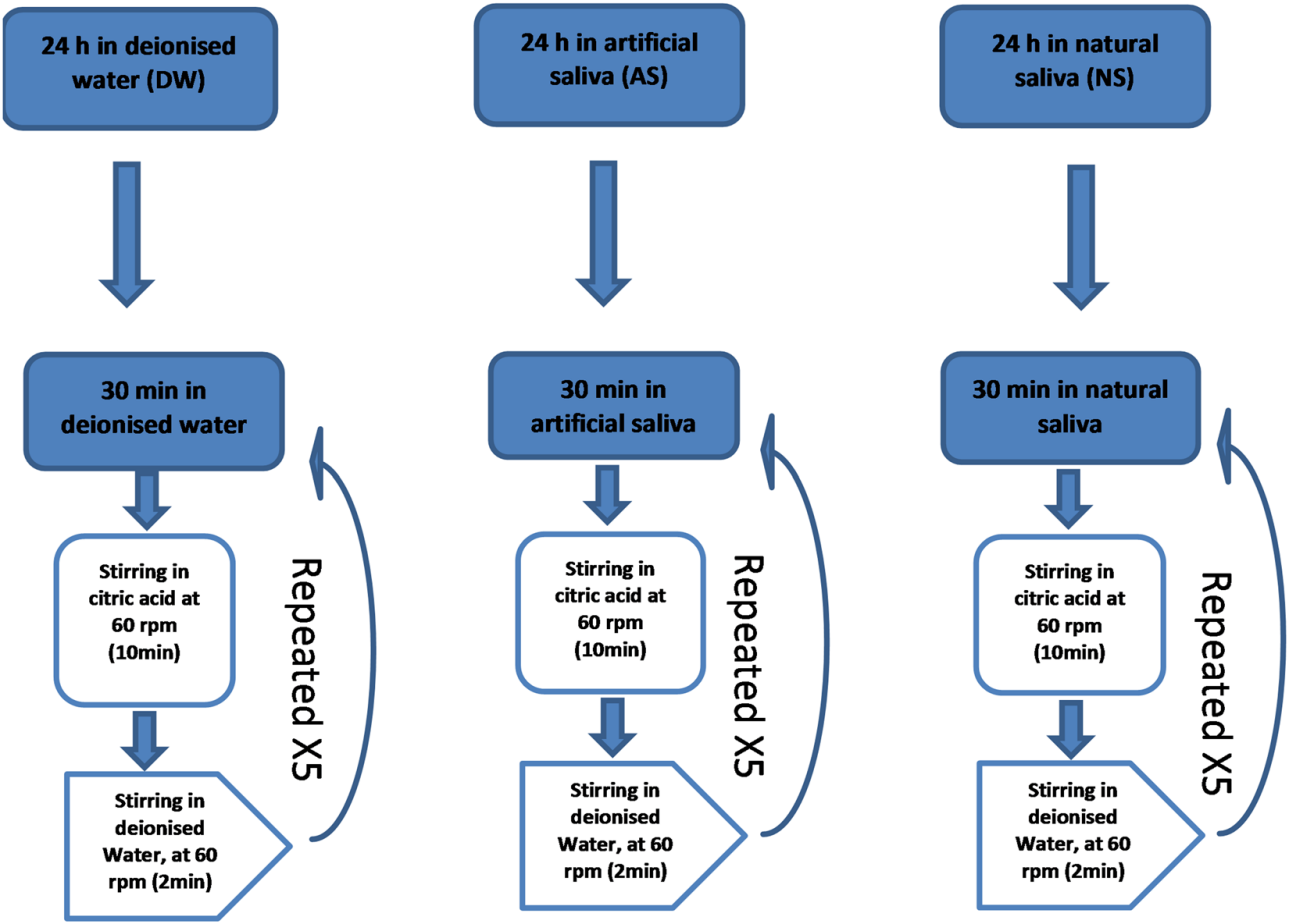
A flowchart representation of the pellicle formation and erosion cycle protocol.

The study consisted of three groups of 10 specimens each: natural saliva (NS), artificial saliva (AS) and deionised water (DW). Specimens were immersed in the corresponding solution for 24 h, followed by a further 30 min in the same solution prior to exposure to a 10-min acid erosion. Immersion of the specimens for 30 min in the corresponding solution followed by the 10-min acid erosion was repeated 5 times for each group, as shown in Fig. 1. The 10-min acid erosion consisted of 80 ml 0.3% citric acid (Sigma Aldrich, Saint Louis, MO 63103, USA), 0.02 M, pH = 3.2, at 22 °C ± 1, agitated with an orbital shaker (Bibby Scientific, Staffordshire, UK) at 60 rpm, followed by a 2-min rinse in 80 ml of deionised water, again under agitation with an orbital shaker set at 60 rpm for a final 2 min.

*In vitro* pellicles were formed as described in the literature^25, 28, 33–39^ through the immersion of specimens in natural saliva over a specific time period. After immersion for 24 h in saliva, specimens were stored un-agitated overnight at 22 °C ± 1.

Specimens were air-dried for 24 h, after which the tape was carefully removed to prepare specimens for profilometric measurement, microhardness testing and atomic force microscopy (AFM) assessment. Step height was measured after five cycles of erosion using a surface non-contacting profilometer (SNCP) (Taicaan XYRIS 2000, Taicaan™ Technologies Ltd., Southampton, UK). SNCP assessment used a white laser light with a 7 µm spot size to scan over the reference and eroded areas of an enamel specimen of 6 mm × 3 mm, X/Y area, ensuring that equal widths of reference and eroded areas were captured. The white laser light scanned the specimen surface line by line in a raster pattern with a single line profile of data points recorded on the x-axis, creating a set of multiple parallel profile measurements, 10 µm apart. Ten randomly selected step height measurements were taken from each specimen, and the average of these readings was calculated to establish the mean step height in μm. The amount of tissue lost was quantified as the height from the reference area to the bottom of the eroded area using surface analysis software (Boddies 2D v1.4 TaiCaan Technologies Ltd., Southampton, UK).

Surface microhardness was measured at baseline (before testing) (SMHb) using a Knoop microhardness tester (Duramin 2, Struers, Germany). Only specimens with an average baseline surface microhardness range between 300 KHN and 400 KHN were selected for the experiment^40^. The value of each specimen was determined by the average of five indentations, at 100 µm intervals from each other, under a load of 100 g and a dwell time of 10 seconds. The value of each indentation was determined by measuring the length of that indentation with an optical analysis system and was then transferred to a computer in Knoop units (KHN). SMH values before and after 24-h immersion in solution were calculated to assess the effect of the solution alone on the microhardness values prior to the erosion cycle. SMH values after 24-h immersion in solution were selected as the baseline (SMHb) and after testing (SMHe) for calculating the surface microhardness change (SMHC) after five cycles of erosion. The SMHC = (SMHb – SMHe) was then calculated.

Atomic force microscopy (Nanowizard 3, JPK Ltd, Cambs., UK) was undertaken in Quantitative Imaging™ mode (QI) in air with Si_3_N_4_ high spring-constant cantilevers (ACTA, AppNano, USA) calibrated using the dedicated JPK software spring constant measurement procedure. QI mode was chosen because it allows a low contact-force to be used, reducing the potential for surface damage during imaging. Three specimens were randomly selected from each group (n = 9) for AFM analysis. On each specimen, images were obtained at three randomly selected areas in both the eroded and the non-eroded region. All images were obtained over a 50 × 50 μm^2^ area at a rate of 0.9 Hz with a resolution of 256 × 256 pixels (256 lines/sample), using a maximum contact force of 2.5 N. From the QI images, the number average area roughness (Sa) was measured for each scanned area.

### Statistical analysis

Data for profilometry, microhardness, and roughness were analysed using SPSS vers22 for normality using histograms, boxplots and Shapiro-Wilks tests. The profilometry, microhardness and stiffness data were normally distributed, whereas the area roughness data required a log transformation, after which they were normally distributed. Data are therefore described as the means and standard deviations. Paired t-tests were used to compare the mean microhardness difference between the SMH values before and after 24-h treatment. Two-way ANOVA was used to determine whether statistically significant differences existed between the means of groups. A post hoc Bonferroni test was used to determine which means were significantly different from each other. The mean difference was considered to be significant at a p value < 0.05.

## Results

### Step height

**Figure 2:**
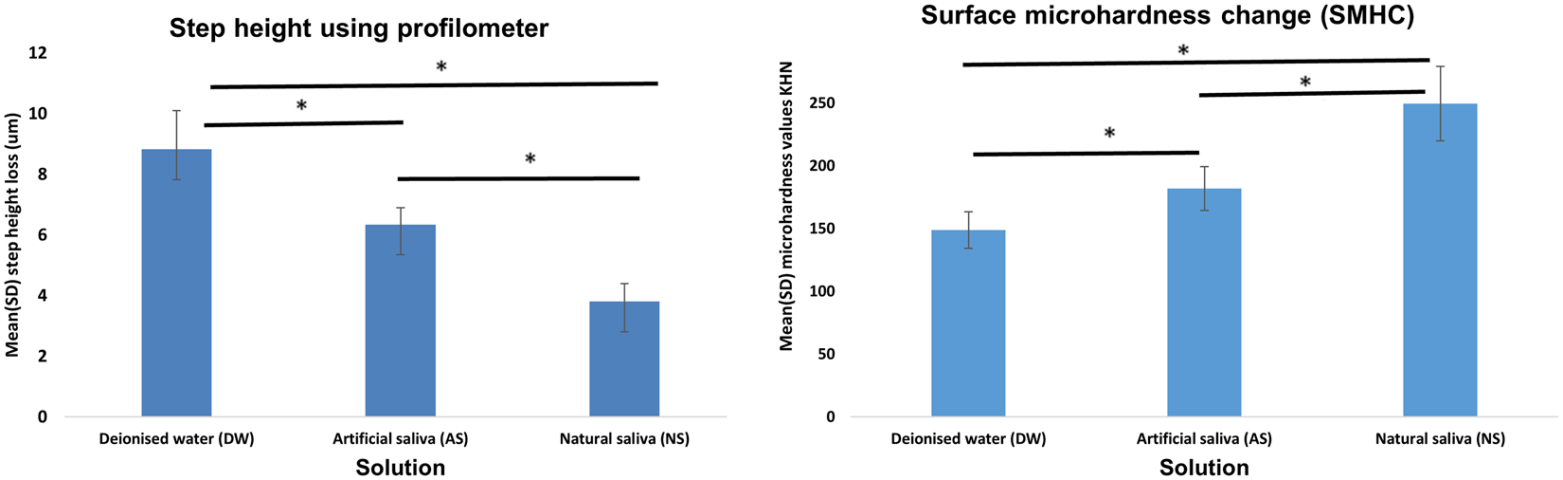
Mean (SD) step height (µm) and surface microhardness change (SMHC) for three groups according to the solution used (NS, AS, DW). Significant differences were observed between all groups in both measurements (step height and surface microhardness <0.0001). Asterisks indicates significant differences between the groups (Paired t tests and two way ANOVA test followed by Post Hoc Bonferroni test, p < 0.05).

Figure 2 shows the mean [standard deviation (SD)] step heights (µm) for the three experimental groups. The NS group had significantly lower step height [3.80 (0.59)] than the AS group [6.34 (0.55) p < 0.001] and DW group [8.80 (1.28) p < 0.0001]. The AS group had a significantly lower step height than the DW group (P < 0.0001).

### Surface microhardness change (SMHC)

Figure 2 also shows the mean (SD) SMHC for three experimental groups. The NS and AS groups had a significantly greater SMHC [249.4 (29.56) KHN and 181.87 (17.48) KHN, respectively] than the DW group [148.82 (14.68) KHN], and the NS group had a significantly greater SMHC than that of the AS group (P < 0.0001). Interestingly, immersing the enamel specimens in NS and AS prior to the erosion cycle resulted in significant SMHC just by incubation in these solutions without any acidic challenge (data shown in the appendix). The mean (SD) SMH values of enamel specimens before 24-h immersion in NS and AS [343.63 (12.21) KHN and 354.09 (15.71) KHN, respectively] exhibited significant reduction after 24-h immersion in NS and AS [315.72 (11.74) KHN p < 0.0001 and 321.79 (10.49) KHN p < 0.001, respectively]. The mean (SD) SMH value at baseline [345.32 (15.29) KHN] was not significantly different from that after 24-h immersion in DW [329.00 (19.31) KHN p < 0.05].

### Atomic Force Microscopy (AFM) analysis

**Figure 3:**
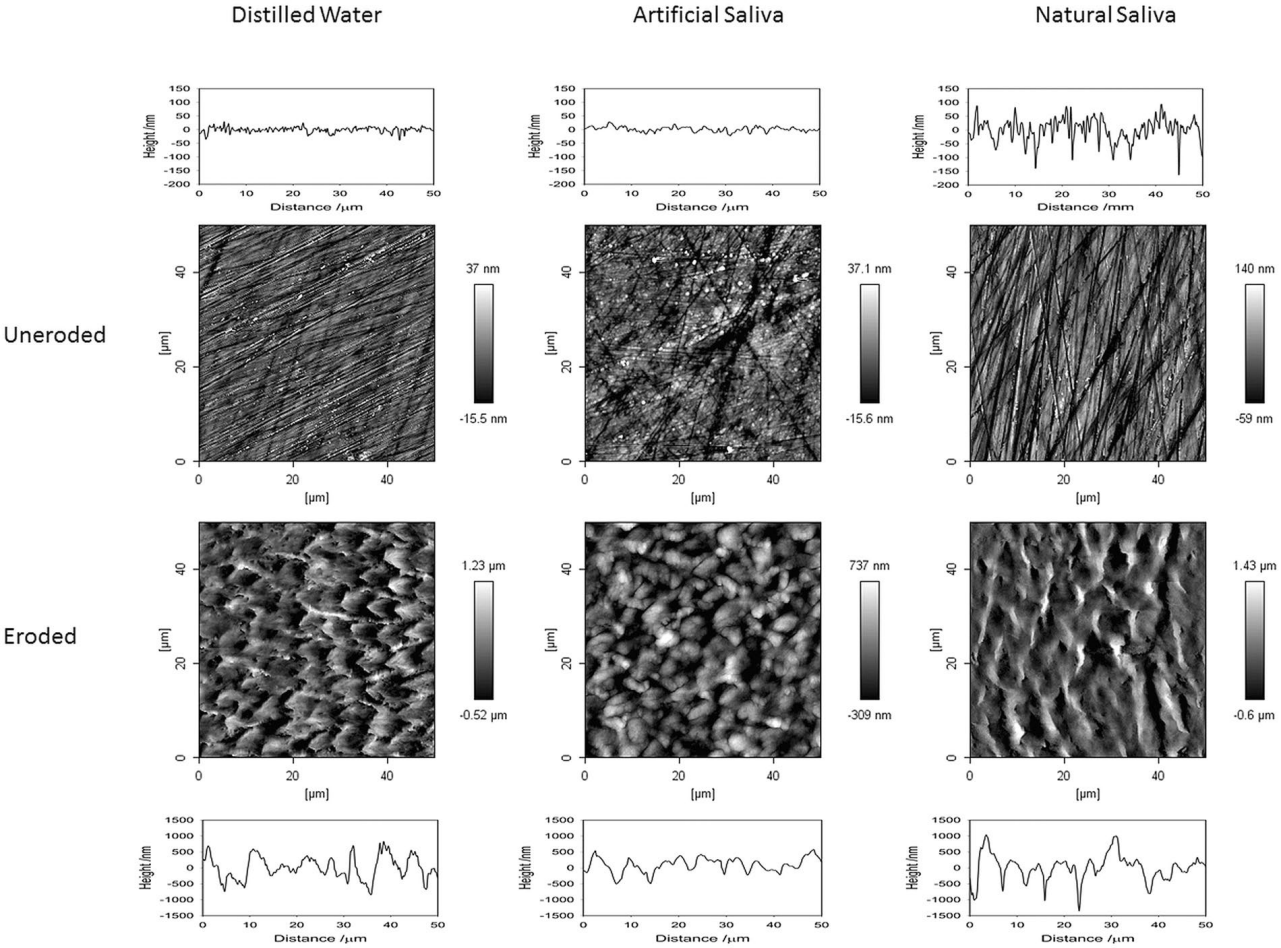
Typical AFM micrographs and 2D line profiles for non-eroded and eroded areas for all three storage solutions (NS, AS, DW).

**Figure 4:**
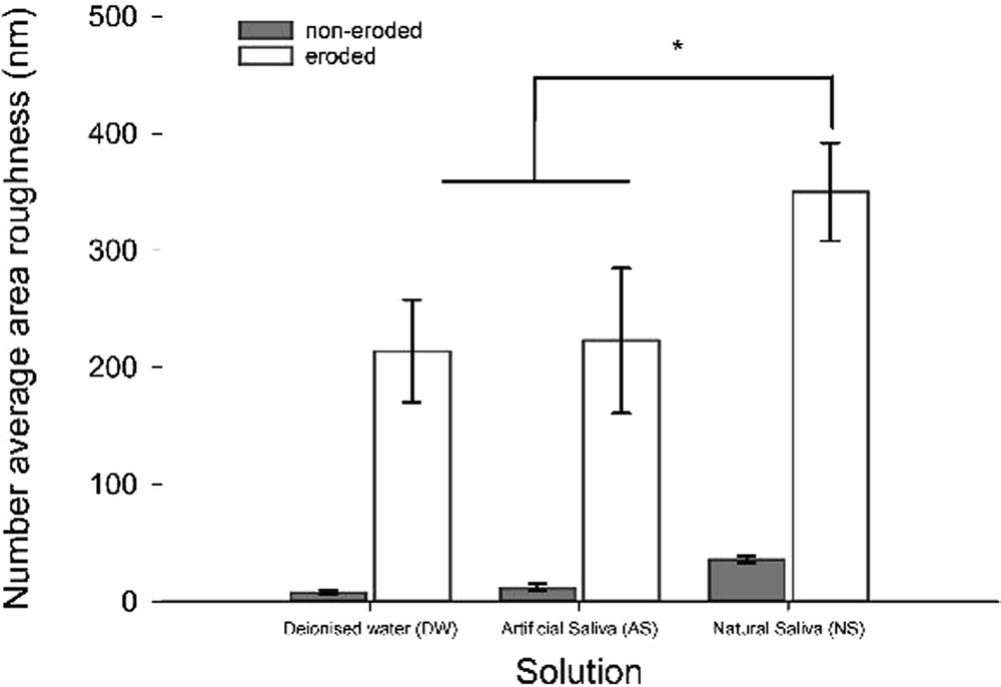
Mean (SD) areal roughness for the three groups according to the solution used (NS, AS, DW) for the non-eroded and eroded areas. Significant differences were found between the eroded and non-eroded surfaces for each solution (P < 0.001, not shown for clarity). Significant difference was found between the NS and the two other surface (indicated by asterisks, two way ANOVA with Bonferroni post hoc test, P < 0.001).

Typical AFM micrographs, together with example 2D profiles, for a randomly selected specimen from each group are shown in Fig. 3, with the average area roughness values for all specimens summarised in Fig. 4. The topography images for all non-eroded areas appeared similar, exhibiting surface scratches typical of a mechanically polished surface with features generally within the 100-200 nm range. Although the roughness of these non-eroded surfaces was low (below 50 nm), significant differences were found between the roughness of all the non-eroded areas, with the NS specimens being the roughest (P < 0.05). By contrast, the eroded areas on the specimens exhibited a markedly different appearance to the non-eroded areas, with the surface scratches no longer visible and significantly greater roughness when compared to the non-eroded areas (P < 0.001).

The appearance of the eroded areas was also different depending on the solution the specimens had been treated with. The specimens immersed in DW exhibited the characteristic lock-and-key appearance of enamel prisms indicative of an eroded enamel surface^41^. Specimens immersed in AS exhibited very different surface topography, characterised by a much narrower height range and less well-defined prism-like structures compared with those stored in DW. Finally, the specimens immersed in NS appeared to have very rough surfaces, characterised by steep peaks and sharp valleys, potentially showing the early stages of erosive wear, with some prism-like structures beginning to appear. These differences in appearance were mirrored in the roughness data for the eroded areas, with the NS specimens having a significantly higher roughness than specimens stored in the other two solutions (Fig. 4, AS and DW, P < 0.001). No significant difference was found between the roughness for specimens immersed in either AS or DW (P > 0.05).

## Discussion

The inclusion of saliva in dental erosion models is often overlooked or omitted due to perceived difficulties in collection, storage and analysis. The results of this study indicate that immersion of enamel specimens in NS for 24 h prior to acid exposure offered the best protection against step height formation [3.80 (0.59) µm], but with a greater Knoop microhardness change [249.4 (19.56) KNH] compared to AS [6.34 (0.55) µm and 181.87 (20.48) KHN, respectively] and DW [8.80 (1.28) µm and 167.12 (15.68) KHN, respectively]. This result is clearly important, as it demonstrates significant differences at and below the surface of the enamel if pre-treated with NS (proteins and ions) or AS (ions) compared to DW (neither proteins nor ions). The results clearly show the protective nature of NS on bulk tissue loss compared to AS and DW. The process of softening may have been due to the effect of the AEP on the dissolution process by which protons destroy the crystal matrix and supports organic structures, slowing the rate of the erosion process. Another possible explanation could be that the protein complexes on the enamel surface decrease the remineralising effect, creating a porous eroded subsurface^21^. Softened enamel may be more susceptible to abrasion from the soft tissues, mucosa and opposing teeth, as well as extrinsic abrasion by toothbrushes and toothpastes. However, the softened enamel is also amenable to possible remineralisation and, therefore, together with the reduction of bulk tissue loss, the softening could also be viewed as protection. Although our experimental model is designed to examine the demineralisation process of dental erosion rather than remineralisation, the AEP is possibly modifying the ion exchanges that occur during acidic challenges and plays a role in both demineralisation and remineralisation. Clearly, the AEP is not working as a barrier to the protons, as considerable softening occurred in all groups, especially the NS group, suggesting protons had permeated the AEP. Thus, it is more likely that the AEP modifies ion movements (protons in, calcium and phosphate out), helping to maintain a high calcium concentration adjacent to the tooth.

Protection against enamel erosion was provided by NS and AS, and the mineral components may have provided some protection against erosion, which supports previous findings^25, 28, 42^. Using transverse microradiography, Amaechi *et al*.^28^ investigated the remineralisation effect on bovine incisors by natural and artificial saliva after 1 h of immersion in orange juice^28^. They observed significant remineralisation using mean mineral loss and lesion depth analysis following exposure to artificial saliva compared to deionised water. Ganss *et al*.^43^ also reported that a layer of minerals can be formed on the enamel surface that would be dissolved when acid attacks enamel, reducing the erosion of underlying enamel surface^43^. Other studies using longer periods of immersion in artificial saliva have shown rehardening of eroded surfaces^25, 28, 31, 44^. Using a combined profilometric measurement with ultrasonication, Eisenburger *et al*.^31^ showed that when enamel specimens were exposed to artificial saliva for 24 h following an erosion cycle of 0.3% citric acid at pH 3.2 for 2 h, complete rehardening of enamel surfaces was observed by measuring softened surface depth before and after ultrasonication^30^. The significant reduction in microhardness indentations after 24 h of treatment with NS and AS could be due to the mineral deposition, which may be influenced by the presence or absence of proteins, as evidenced by the different effects undergone by NS versus AS. Some proteins within the AEP possess phosphate groups such as statherin, and proline-rich proteins have been observed to adhere rapidly and strongly to the enamel crystals, which may potentially play a role in the regulation of calcium phosphate homeostasis^45, 46^. Ionic calcium and phosphorus concentration help maintain the integrity of enamel crystals by balancing the calcium in the calcium phosphate of the tooth against the surrounding fluids. Adding mucin to AS has also been found to be as effective as NS to significantly improve the erosion-inhibiting properties of the AEP, more so than AS and DW^25^. For *in vitro* studies of erosion, NS is commonly replaced by AS which can be presented in three general formulations. These are electrolytes with carboxymethylcellulose (CMC)^28, 30^, electrolytes with mucin^25, 47^ or electrolyte only^31^. Some of these formulations have been found to yield a remineralising effect to eroded enamel^28, 31, 48, 49^ whereas other studies suggest that AS formulations may be detrimental for the remineralisation process and can significantly increase the progression rate of the erosive tooth wear lesion^20, 25, 50^.

Although an AEP can form within minutes, a longer immersion time of enamel specimens in saliva (24 h) in solution was selected in the present study. Although one study demonstrated that 24-h-formed pellicles showed the same protective effect as 7-day-old pellicles^14^, two other studies suggested that several-day-old pellicles provided greater protection against demineralisation^44, 45^. Amerongen *et al*. (1986) demonstrated that protection against demineralisation was improved with pellicle formation time of up to 6 days^51^. Featherstone *et al*.^52^ also found a good linear relationship between the time of pellicle formation, up to 7 days, and mineral loss^52^. Although AEP formed over shorter periods may offer similar protection as those formed over a longer period, they are more soluble and may dissolve more quickly after an acid challenge^53^.

Surface non-contact profilometry (SNCP) and surface microhardness (SMH) testing were used in this study to provide a broad range of information on the surface change of eroded enamel surfaces. SNCP is considered the ‘gold standard’ technique for *in vitro* tooth-wear measurements^54^. SMH is useful for measuring early surface softening but not suitable for measuring erosive tooth wear when tissue loss has occurred^3, 55^. Therefore, SNCP is the suitable technique for measuring bulk tissue loss. A combination of SNCP and SMH has been previously used to assess the tissue dissolution and surface softening of enamel, respectively^56^.

AFM images confirm the results from SNCP and SMH; that is, the mechanisms of erosive tooth wear are different between enamel surfaces immersed in different solutions. Although the data in the literature on surface texture are still contradictory, it is generally understood that erosive challenges increase enamel roughness to a certain degree before smoothing of the surface occurs^57^. In this study, the AFM images of enamel specimens immersed in DW exhibited the characteristic scallop-shell surface indicative of erosive wear of both prismatic and inter-prismatic enamel^41^. The specimens immersed in natural saliva appeared to show the early stages of erosion, with some prism-like structures potentially appearing; however, this effect was significantly less clear than with the specimens immersed in deionised water. These surfaces were found to be the roughest of all specimens, with the centres of prisms being lost while the peripheral tissue remained relatively undamaged, leading to the sharp peak-and-troughs appearance seen in the 2D profile. This behaviour has been reported previously^41^ and is typical of the type-1 etching pattern first proposed by Silverstone *et al*.^58^. The enamel specimens immersed in artificial saliva showed a different behaviour, which, although different to the non-eroded surfaces, was considerably flatter than either of the specimens immersed in natural saliva or deionised water, in which their prism-like structures were far harder to define. This result seems to show a difference in the mechanism of erosive wear when specimens have been immersed in artificial saliva compared to natural saliva, something that, to our knowledge, has never been shown before.

AFM is becoming increasingly commonly used in the study of dental erosion due to the potential to combine high resolution imaging with surface profilometry. Consequently, images can be obtained at a comparable resolution to scanning electron microscopy, from which roughness measurements can be easily obtained. Within the dental literature, the most commonly reported roughness parameters are either Ra (number average roughness) or Rq (root mean square roughness)^59^ measured from 2D profiles. In this study, we measured the areal equivalent to Ra, namely, Sa, which allowed us to obtain a more complete understanding of the surface textural changes caused by each of the storage solutions compared to using Ra alone.

This study has provided us with important information on the varied effects of the pellicle on the enamel surface with and without acid erosion, which may be determining the *in vivo* pellicle response to acid challenges. However, the findings of *in vitro* studies, such as the present study, should be interpreted with caution. It is difficult to say whether the significant difference in the protection from erosion offered by the *in vitro* 24-h-formed pellicle is directly relevant to the clinical situation. The changes and degradation of pooled saliva due to the collection, storage, and cycling, such as CO_2_ evaporation, may have altered the salivary protective properties^21, 27, 34^. *In situ*/*in vivo* clinical studies are needed to demonstrate the long-term effectiveness of formed pellicles against erosion. In addition, as our *in vitro* model has assessed solutions containing proteins and ions (NS) and ions only (AS) compared to that containing neither proteins nor ions (DW), it might have been advantageous to include a protein-only control of dialysed saliva as others have done^60^. Moreover, the susceptibility of the pooled saliva to proteolytic activity does not mimic that of the fresh saliva in the oral cavity. It would therefore be of interest for future studies to investigate the effect of the proteolytic salivary enzymes on the 24-h-formed pellicle in relation to erosive wear. The remaining softer structure after the 24-h-formed pellicle could open up new horizons of interesting research lines on erosive wear therapies and prevention.

## Conclusions

Twenty-four hours of exposure to natural saliva offered better protection against erosive wear compared to artificial saliva and deionised water, manifested by less step height formation but greater microhardness change and rougher surface. This result highlights the importance of using natural saliva in future laboratory studies. This study also demonstrates that the combined presence of salivary minerals and proteins in *in vivo* dynamics may influence the response of the pellicle to acid challenges, indicating that the saliva status in individuals is an important clinical parameter to consider in protection against erosive tooth wear.

## Electronic supplementary material

Supplementary Information
