# Supplementary material for: The presence of acquired enamel pellicle changes acid-induced erosion from dissolution to a softening process
Source: Sci Rep. 2017 Sep 7;7:10920. doi: 10.1038/s41598-017-11498-1 (PMC5589892; doi:10.1038/s41598-017-11498-1)
Supplement: Supplementary file 1 — Supplementary Information [file 41598_2017_11498_MOESM1_ESM.pdf]

The presence of acquired enamel pellicle changes acid-induced erosion from a dissolution- to a softening- process

Mahdi Mutahar[1], Guy Carpenter[1], David Bartlett[2], Matthew German[3], Rebecca Moazzez[1]

Appendix

Surface microhardness (SMH) values before and after immersing enamel specimens in solutions before exposure to citric acid.

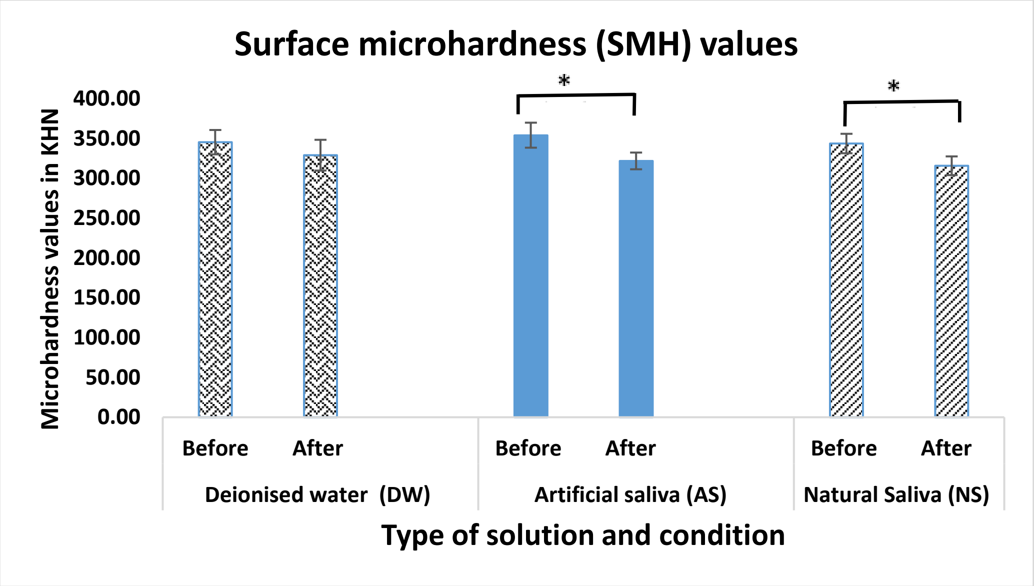

Mean (SD) surface microhardness (SMH) values in Knoop microhardness units (KHN) for three groups according to the solutions used (NS, AS, DW). Each group (n=10) is represented by two bars: before and after 24 h immersion in the pertaining solution (no acid exposure). NS and AS groups exhibited significant SMHC whereas DW group did not. Paired t tests, $p < 0.05$. Asterisks indicate significant differences.
